# Co-infection with *Trypanosoma cruzi *protects mice against early death by neurological or pulmonary disorders induced by *Plasmodium berghei *ANKA

**DOI:** 10.1186/1475-2875-6-90

**Published:** 2007-07-09

**Authors:** Claudia M Egima, Silene F Macedo, Gisela RS Sasso, Charles Covarrubias, Mauro Cortez, Fernando Y Maeda, Fabio T Costa, Nobuko Yoshida

**Affiliations:** 1 Departamento de Microbiologia, Imunologia e Parasitologia, Universidade Federal de São Paulo, São Paulo, SP, Brasil; 2 Departamento de Morfologia, Universidade Federal de São Paulo, São Paulo, SP, Brasil; 3 Departamento de Parasitologia, Instituto de Biologia, Universidade Estadual de Campinas, Campinas, SP, Brasil

## Abstract

**Objective:**

The objective of this study was to investigate whether the infection of C57BL/6 mice by *P. berghei *ANKA, which causes severe malaria, was modulated by co-infection with *Trypanosoma cruzi*.

**Methods:**

Groups of C57BL/6 mice were infected either with *P. berghei *ANKA, *T. cruzi *strain G, or with both parasites. The presence of parasites was checked by microscopic examination of blood samples. Symptoms of neurological or respiratory disorders, as well as mortality, were registered. Breakdown of the blood brain barrier was determined by injecting the dye Evans blue. Histological sections of the lung were prepared and stained with hematoxilin-eosin.

**Results:**

All mice infected only with *P. berghei *ANKA died within 7–11 days post-infection, either with symptoms of cerebral malaria or with respiratory abnormalities. The animals co-infected with *T. cruzi *strain G survived longer, without any of the referred to symptoms. Protection against the early death by severe malaria was effective when mice were given *T. cruzi *15 days before *P. berghei *inoculation. Breakdown of the blood brain barrier and extensive pulmonary oedema, caused by malaria parasites, were much less pronounced in co-infected mice. The degree of protection to severe malaria and early death, conferred by co-infection with *T. cruzi*, was comparable to that conferred by treatment with anti-CD8 antibodies.

**Conclusion:**

Co-infection with *T. cruzi *protects C57BL/6 against the early death by malaria infection, by partially preventing either the breakdown of the blood brain, and cerebral malaria as a consequence, or the pulmonary oedema.

## Background

Multiple infections by parasitic agents that cause diverse clinical manifestations occur frequently and increase or decrease of overall pathogenic impact can be influenced by synergistic or competitive interactions between parasite species [1,2]. Malaria is prevalent throughout tropical regions where concomitant infections occur frequently. Double infections with *Plasmodium *spp. and *Ascaris lumbricoides*, or triple infections with the two parasites plus *Trichuris trichiura*, without synergism or antagonism among parasites, have been reported in Zaire [3]. Concomitant parasitism by agents of malaria and lymphatic filariasis, with no indication of interaction between the two infections, has been detected in India and in Guyana [4,5]. On the other hand, it has been found that Senegalese children lightly infected with *Schistosoma haematobium *had lower *Plasmodium falciparum *densities, suggestive of negative interactions between both parasites [6]. It has been pointed out that, if worms have in fact deleterious effect on malaria, treatment of helminthic infection would offer an affordable and effective means to roll back malaria [7]. Experiments in mice have shown that malaria-filaria co-infection causes more severe anaemia and loss of body mass than *Plasmodium chabaudi *malarial infection alone [8], and that concomitant *P. chabaudi *and *Schistosoma mansoni *infections increase malaria parasitaemia and suppress spleen cell proliferative and Th2 responses to *S. mansoni *soluble egg antigen [9]. Using *Plasmodium berghei *ANKA, which causes lethal cerebral malaria in C57BL/6J mice, Legesse *et al*. [10] found that superinfection with *S. mansoni *enhanced malaria parasite development, increasing parasitaemia and mortality.

As regards the mixed infection by malaria parasite and another protozoan, the data are sparse. The possibility, for instance, of co-infection with *Trypanosoma cruzi*, the agent of Chagas' disease, has not been examined. In Brazil, as there has been an increase in cases of Chagas' disease in the Amazon, where malaria prevails, recommendations have been made to include the microscopic analysis of blood smears for direct search of *T. cruzi *in patients with fever in the routine survey for malaria parasites [11]. Thirty years ago, Krettli [12] reported that in double infection with *P. berghei *NK65 and *T. cruzi *about 40% of mice chronically infected with *T. cruzi *relapsed to the acute phase when inoculated with *P. berghei*, while some decrease in *P. berghei *parasitaemia was observed. Since then, the question has not been further addressed. This study aimed at investigating if, and to what extent, the agents of malaria and Chagas' disease exerted their effects to each other's course of infection. To that end, *P. berghei *ANKA, which produces cerebral malaria in C57BL/6 mice, and a *T. cruzi *strain from the Amazon, which produces subpatent infection, were used.

## Methods

### Parasites and infection of mice

*P. berghei *ANKA was conserved as stabilates of 10^7 ^parasitized erythrocytes in liquid nitrogen. When needed, the parasites were defrosted and injected intraperitoneally into C57BL/6 mice and seven days later these served as the inoculum to infect mice. C57BL/6 mice bred in the animal facility (CEDEME), at Universidade Federal de São Paulo, were used throughout. All procedures and experiments conformed to the regulations of the institutional Ethical Committee for animal experimentation. In all assays, mice were infected with *P. berghei *by inoculating intraperitoneally 10^6 ^parasitized erythrocytes. Parasitaemia was monitored by reading Giemsa-stained blood smears. The *T. cruzi *G strain, isolated from an opossum in the Brazilian Amazon [13] was maintained cyclically in Swiss mice and in liver infusion tryptose medium. Infective metacyclic trypomastigotes from cultures at the stationary growth phase were purified by passage through DEAE-cellulose column, as described earlier [14]. For standard co-infection experiments, C57BL/6 mice were inoculated intraperitoneally with 10^6 ^*T. cruzi *metacyclic forms, and 15 days later they received 10^6 ^erythrocytes parasitized with *P. berghei *ANKA. The *T. cruzi *parasitaemia was checked by counting the parasites in 5 μl fresh blood collected from the mouse tail, under phase-contrast microscope.

### Injection of Evans blue into mice and removal of the brain

The dye Evans blue was prepared as a 1% solution in PBS and each mouse received 0,2 ml through intraorbital route. When *P. berghei*-infected mice showed signs of neurological disorder, such as deviation of the head, convulsion and paralysis, they were given the dye and one hour later the brain was removed. Each time a *P. berghei*-infected mouse received the dye, a co-infected mouse and a mouse infected only with *T. cruzi*, were injected with the dye and their brain collected one hour later. The brains were stored at 4°C in a petri dish with PBS, and at the end of the experiment their images were recorded with a digital camera.

### Preparation of histological sections of mouse lung

Experiments in which *P. berghei*-infected mice presented respiratory disorders, right after death their lungs were collected and fixed in 10% neutral buffered formalin for 24 h. Afterwards, the organs were gradually dehydrated in ethanol solution at different concentrations, followed by immersion in xylol, and then embedded in paraffin. Serial sections, 5 μm thick, were prepared and stained with hematoxylin-eosin. Equivalent numbers of symptomless mice, co-infected with *T. cruzi*, were also killed and their organs processed as above.

### Treatment of mice with anti-CD8 antibodies

Mice infected with *P. berghei *ANKA only, or co-infected with *T. cruzi*, were treated with anti-CD8 antibodies (0.3 ml/mouse of ascitic fluid from clone TIB 21O by intraperitoneal injection), on days 0 and 5 of malaria infection.

## Results and Discussion

### Co-infection with *T. cruzi *protects mice from early death by *P. berghei *ANKA

**Figure 1:**
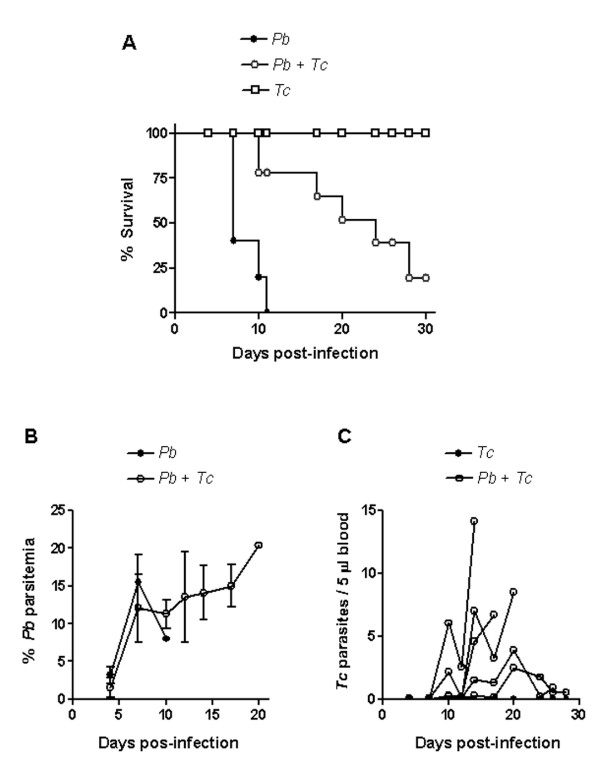
Mortality and course of infection in mice infected with *P. berghei *ANKA and/or *T. cruzi *G strain. Of three groups of C57BL/6 mice from the same batch, two were inoculated with *T. cruzi *metacyclic forms. Fifteen days later, *P. berghei*-parasitized erythrocytes were given to the uninfected (n = 6) and to one of the *T. cruzi*-infected group (n = 7), whereas one group remained infected with *T. cruzi *only (n = 6). A) Note the increased survival time in co-infected mice, as compared to the group infected with *P. berghei *only. B) Parasitaemia of *P. berghei *is expressed in percentage of parasitized erythrocytes and the values indicate the means ± SD. C) *T. cruzi *parasitaemias of individual mice are shown. Shown are the representative data from a set of experiments.

*T. cruzi *G strain was used because it produces a non lethal subpatent infection in mice, even when the animals are inoculated with a number of parasites as high as 8 × 10^6 ^[13]. C57BL/6 mice were separated in three groups, two of which were inoculated with 10^6 ^metacyclic forms of *T. cruzi *G strain. Fifteen days later, one uninfected and one *T. cruzi*-infected group were inoculated with 10^6 ^erythrocytes parasitized with *P. berghei *ANKA. Four days later, monitoring of mortality and course of infection was initiated. Fifty percent of *P. berghei*-infected mice were dead by day 7, and by day 11 there was not a single survivor, whereas co-infected mice survived longer, 50% of them being alive by day 17 (Figure 1A). A five-week *T. cruzi *infection was less effective than a two-week infection in protecting against early death by severe malaria. In experiments in which *T. cruzi *and *P. berghei *were inoculated simultaneously, or in which *T. cruzi *infection started one week before malaria infection, no protection was achieved.

Parasitaemia of *P. berghei *reached ~16% at the peak in mice with single infection, and around 20–30% in doubly infected animals (Figure 1B). On the other hand, *T. cruzi *blood forms, which are not detectable in a single infection unless the animals are immunosuppressed by irradiation or by treatment with cyclophosphamide, could be visualized in most animals co-infected with *P. berghei *(Figure 1C). This finding is compatible with the immunosuppressive effect induced by malaria infection [15-17]. Although rendered positive by co-infection, *T. cruzi *parasitaemia levels were very low as compared to those of virulent strains [13].

### Symptoms of cerebral malaria are absent and breakdown of brain blood barrier is less pronounced in mice co-infected with *T. cruzi*

One of the symptoms of C57BL/6 mice with *P. berghei *ANKA is the neurological disorder leading to paralysis, and ultimately to coma and death, characteristic of cerebral malaria. In the experiment described in Figure 1, the majority of mice infected with *P. berghei *presented signs of neurological damage before dying. In repeat experiments, such abnormality was not seen in mice co-infected with *T. cruzi*.

**Figure 2:**
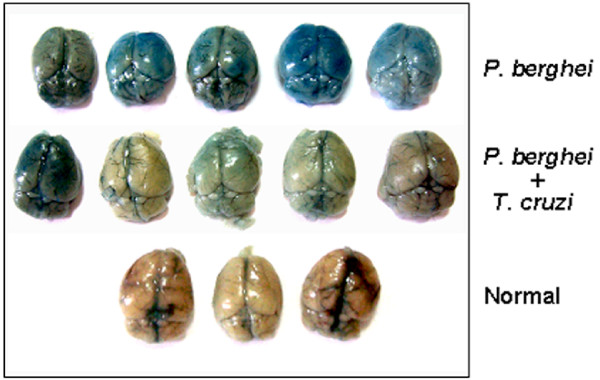
Breakdown of blood brain barrier in mice infected with *P. berghei *ANKA or co-infected with *T. cruzi*. C57BL/6 mice were separated in 4 groups, two of which were inoculated with *T. cruzi *G strain. Fifteen days later, one uninfected (n = 5) and one *T. cruzi*-infected group (n = 5) were inoculated with *P. berghei *ANKA, whereas one group remained uninfected (n = 3). When *P. berghei*-infected mice showed signs of neurological disorder, they were given the Evans blue and 1 h later the brain was removed. At this time, the corresponding number of mice in other groups received the dye and the brain was collected 1 h later. Note the difference of brain color in the three groups. The brains of the group with single *T. cruzi *infection (n = 5) (not shown) were indistinguishable from the normal mice.

Cerebral malaria in mice infected with *P. berghei *ANKA is accompanied by mononuclear cell infiltration, haemorrhage and cerebral endothelial damage, this damage presumably being one of the causes of the disruption of the brain blood barrier [18]. Experiments were performed to detect the destruction of the blood brain barrier, by injecting mice with the dye Evans blue. C57BL/6 mice were separated in four groups, two of which were inoculated with metacyclic forms of *T. cruzi *G strain. Fifteen days later, one uninfected and one *T. cruzi*-infected group were inoculated with *P. berghei *ANKA. When *P. berghei*-infected mice showed signs of neurological disorder, they were given the dye and one hour later the brain was removed. At this time, the corresponding number of mice in the co-infected, *T. cruzi*-infected or uninfected control groups, received the dye and the brain was collected one hour later. The higher the blue color intensity of the brain the higher the breakdown of the blood brain barrier. The brains of mice infected with *P. berghei *were colored in deep blue whereas most of those from co-infected mice were light blue (Figure 2). Mice infected only with *T. cruzi *had normal brain colour (not shown), indistinguishable from the normal controls (Figure 2). This result suggests that, by preventing the extensive destruction of the blood brain barrier, co-infection with *T. cruzi *protects against cerebral malaria.

### Co-infection with *T. cruzi *protects mice from extensive pulmonary edema induced by malaria parasites

**Figure 3:**
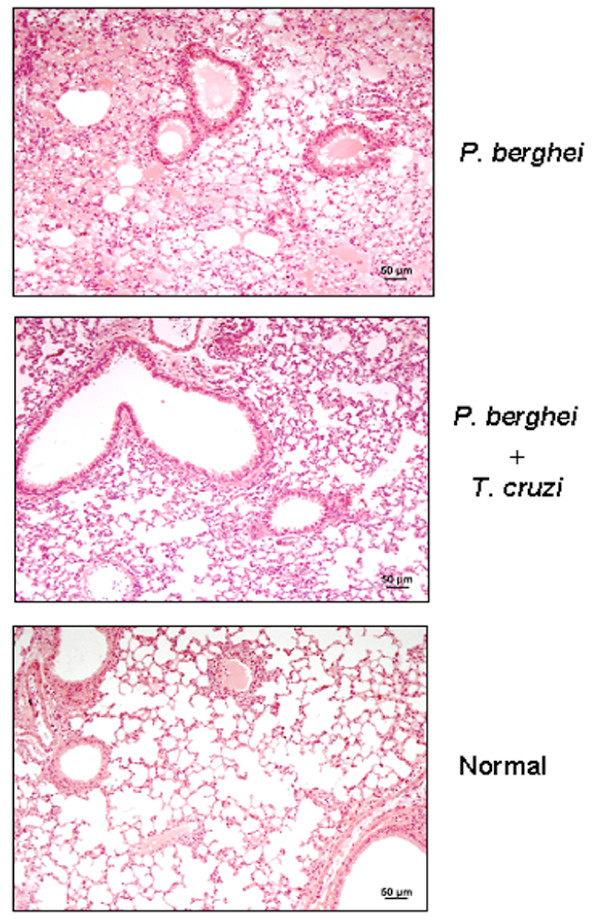
Pulmonary oedema induced in mice infected with *P. berghei *ANKA or co-infected with *T. cruzi*. C57BL/6 mice were separated in three groups, two of which were inoculated with *T. cruzi *G. strain. Fifteen days later, one uninfected (n = 5) and one *T. cruzi*-infected group (n = 5) were inoculated with *P. berghei *ANKA, whereas one group remained uninfected (n = 3). When *P. berghei*-infected mice died from respiratory disorder, their lungs were collected for histological preparations and staining with hematoxilin-eosin. At this time, the corresponding number of mice in the co-infected group had also their lungs removed for histological sections and staining. Uninfected controls were subjected to the same procedure.

Pulmonary involvement occurs in 3–10% of individuals infected with *P. falciparum *and represents the most serious complication of this infection, with lethality of about 70% [19]. In severe *P. falciparum *malaria, the abnormal vascular change was found to be the cause of pulmonary oedema [20]. Pulmonary oedema and changes in clotting and fibrinolysis have been observed during *P. berghei *infection in mice [21]. In some experiments of this study, *P. berghei*-infected mice suffered from respiratory syndrome before dying. This complication appeared around day 6 or 7 after malaria parasite inoculation and by day 10 all mice were dead. No such complications were found in mice co-infected with *T. cruzi*. To determine whether the observed respiratory distress was associated with pulmonary oedema, within five minutes after death the lung of *P. berghei*-infected mice was removed and processed for histological analysis. At the time of removal, abundant liquid was noted, possibly resulting from transfer to the interstitial space and alveoles, as described in *P. falciparum *malaria [19]. No such alteration was found in mice co-infected with *T. cruzi*, which had their lungs collected at the time of death of *P. berghei*-infected animals. Extensive pulmonary oedema was seen in *P. berghei*-infected mice, whereas the cellular infiltration in the lung of co-infected mice was less pronounced (Figure 3).

### Protection to severe malaria and early death conferred by co-infection with *T. cruzi *is similar to that observed in *P. berghei *ANKA-infected mice treated with anti-CD8 antibodies

**Figure 4:**
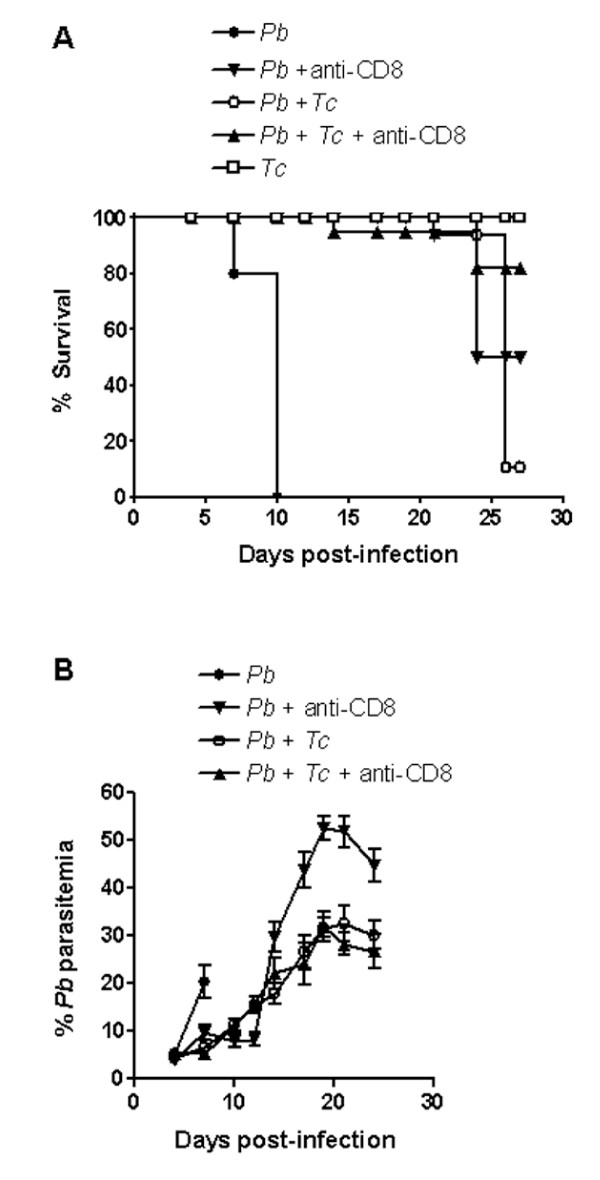
Effect of anti-CD8 antibodies on the mortality and course of infection in mice infected with *P. berghei *ANKA or co-infected with *T. cruzi *G strain. Of five groups of C57BL/6 mice, three were inoculated with *T. cruzi *metacyclic forms. Fifteen days later, *P. berghei*-parasitized erythrocytes were given to two uninfected groups (each n = 5), two *T. cruzi*-infected group (each n = 5), whereas one group remained infected with *T. cruzi *only (n = 5). On day 0 and 5 of malaria infection, one *P. berghei*-infected and one co-infected group were treated with anti-CD8 antibodies. B) Parasitaemia of *P. berghei *is expressed in percentage of parasitized erythrocytes and the values indicate the means ± SD.

According to Belnoue *et al. *[22], CD8^+ ^T cells, which are sequestered in the brain of mice at the time of appearance of neurological symptoms, are responsible for mortality by cerebral malaria. Analysing mice co-infected with two *Plasmodium *species, Voza *et al. *[23] have found complete inhibition of *P. berghei *ANKA-induced cerebral malaria in mixed infection with *Plasmodium yoelii yoeili*, but not with *Plasmodium vinckei *or another line of *P. berghei*. In the protected co-infected mice, the accumulation of CD8^+ ^T cells in the brain vasculature was found to be inhibited. Here the degree of protection against *P. berghei *ANKA infection conferred by anti-CD8 antibodies, and by co-infection with *T. cruzi*, was compared. Both antibody-treated and co-infected mice survived for more than 20 days (Figure 4), without showing any signs of cerebral malaria or respiratory disturbances. Treatment of co-infected mice with anti-CD8 antibodies did not confer further protection.

The mechanism by which co-infection with *T. cruzi *confers protection against severe malaria is unknown. An interesting possibility is that in mixed infection the sequestration of CD8^+ ^T cells in organs or tissues that are targets of *T. cruzi*, such as the heart and skeletal muscles, results in reduced availability of these cell types for localization in the brain. That possibility exists. Predominance of CD8^+ ^T lymphocytes in inflammatory cardiac and skeletal muscles has been observed in mice with acute *T. cruzi *infection [24]. In that study, CD8^+ ^T cells (47.0–58.9%) significantly outnumbered CD4^+ ^cells (9.3–18.6%). As regards the pulmonary oedema in severe malaria infection, it still remains to be determined what induces it. If CD8^+ ^T cells are also involved, the preferential recruitment of these cells to organs that are targets of *T. cruzi *infection, which do not include lungs, might prevent the accumulation of CD8^+ ^T cells in sufficient numbers to produce more severe pulmonary alterations.

This study shows for the first time the modulatory effect of co-infection with *T. cruzi *on malaria infection that otherwise leads to an early death by cerebral malaria or extensive pulmonary oedema. In most regions where malaria is endemic, mixed infections are frequent. Therefore, the varied outcomes of malaria infection may result, at least in part, from positive or negative regulatory effects of other pathogens harbored by the patients.

## Authors' contributions

CME, SFM and NY were involved in all stages of this study. GRSS participated in the preparation and analysis of histological sections. CC and MC participated in the follow-up of parasitaemia in *T. cruzi*-infected and co-infected animals. FYM was involved in the experiments to detect disruption of brain blood barrier. FTC participated in the design of the study. All authors read and approved the final manuscript.
